# Cathelicidin peptide rescues *G. mellonella* infected with *B. anthracis*

**DOI:** 10.1080/21505594.2017.1293227

**Published:** 2017-03-08

**Authors:** Ryan J. Blower, Serguei G. Popov, Monique L. van Hoek

**Affiliations:** School of Systems Biology, George Mason University, Manassas, VA, USA

**Keywords:** antimicrobial peptide, *B. anthracis*, bactericidal, cathelicidin, *G. mellonella*, LL-37, MIC, NA-CATH, sporicidal, waxworm

*Bacillus anthracis* is a Gram-positive spore-forming bacterium that resides in the soil and causes the disease anthrax. *B. anthracis* predominantly affects livestock and is endemic worldwide. The incidence of infection in the developed countries is low. Most commonly, humans become infected due to contact with infected animals or contaminated animal products. Anthrax continues to be of importance to human health as a bio-threat. The infection is typically initiated by one of 3 routes: cutaneous, gastrointestinal and inhalational. Without an appropriate treatment, bacteria from the sites of entry can disseminate to other organs ultimately causing septic shock and death. The inhalation form of anthrax is the most deadly. While antibiotic therapeutics such as ciprofloxacin are available, patients with inhalation anthrax have only a 50% chance of survival,^1^ thus new therapies are needed.

During its vegetative growth phase, *B. anthracis* produces several virulence factors including Lethal and Edema toxins (encoded by plasmid XO1) as well as a poly-γ-D-glutamic acid capsule (encoded by plasmid XO2).^2^ In this work, we used *B. anthracis* Sterne, a strain of anthrax lacking the pXO2. As a result the Sterne strain is strongly attenuated in humans, but displays residual virulence in mice due to the presence of pXO1.

Cationic antimicrobial peptides (CAMPs) are produced as a part of the innate immunity and often display an amphipathic secondary structure. Their overall positive charge allows association with negatively charged outer leaflet of bacterial membrane^3^ while leaving eukaryotic membranes intact.^4^ We previously determined the activity and some mechanistic features for many native and synthetic CAMPs against *Francisella*,^5-8^ *Pseudomonas*,^9^ *Staphylococcus aureus*^10^ and *Burkholderia thailandensis*.^11^ We sought to find peptide-based antimicrobials that could be developed further as potential therapeutics for *B. anthracis* infection, or as compounds to be used in conjunction with antibiotics to increase the overall survival from this infection.

Several CAMPs were previously shown to be effective against the capsulated and non-capsulated *B. anthracis* strains.^12,13^ Interestingly, most of these peptides did not function by directly exerting their antimicrobial action on the bacterial membrane as has been described previously for other AMPs against other bacteria.^14^ For example, protegrin-1 (PG-1), a porcine cathelicidin, functions by altering vegetative outgrowth process.^12^ Theta-defensin retrocyclin displays an immunomodulatory effect to increase macrophage performance^13,15^ and thus achieves a “host-directed” action against *B. anthracis*. In this study, we sought to characterize 8 CAMPs from the cathelicidin family with regard to their antibacterial and sporicidal effects upon *B. anthracis*.

This work introduces an *in vivo* model of testing the activity of CAMPs against *B. anthracis* injected into the hemocoel of the waxworm *G. mellonella*. This provides an opportunity to perform *in vivo* testing in an invertebrate model before moving lead candidates forward to established animal models of infection. Use of *G. mellonella* has been published as an alternative infection model system for a variety of bacterial infections.^8,9,16-24^ Further, many bacterial virulence factors required for bacterial infection in mice were shown to be also required for infection in *G. mellonella*.^25^ This *G. mellonella* model of infection, by injection into the hemocoel, may provide a model of disseminated anthrax infection, as the hemocoel is the circulation system of the insect and contains phagocytic hemocytes. Vegetative cells and spores can be distributed throughout the organism, as in disseminated anthrax.^26,27^ We used *G. mellonella* as the *in vivo* assay to down-select our lead antimicrobial peptides in preparation for future testing in the mouse model of *B. anthracis* infection. This waxworm model allows for testing the antibacterial activity of numerous antimicrobial peptides, which would be impractical in a mouse model.

*B. anthracis* Sterne strain was grown as described in the Supplemental text, and exposed to a panel of CAMPs to test their antimicrobial activity including LL-37 (human cathelicidin); D-LL-37 (D-enantiomer of LL-37)^9^; SMAP-29 (sheep cathelicidin)^28^; PG-1 (porcine protegrin-1)^12^; mCRAMP (murine cathelicidin)^29^; BMAP-28 (bovine cathelicidin)^28^; NA-CATH (Chinese cobra cathelicidin)^5^; and CAP-18 (rabbit cathelicidin).^30^

The EC_50_ antimicrobial assay was performed against vegetative cells (Fig. 1) followed by a standard minimum inhibitory concentration (MIC) determination (Table 1) following our previous publications (Please refer to Supplemental text for detailed methods). Seven of the 8 cathelicidins tested had a similar EC_50_ value to ciprofloxacin (p>0.05), with the exception being BMAP-28, which was 100x less effective.

**Figure 1.**
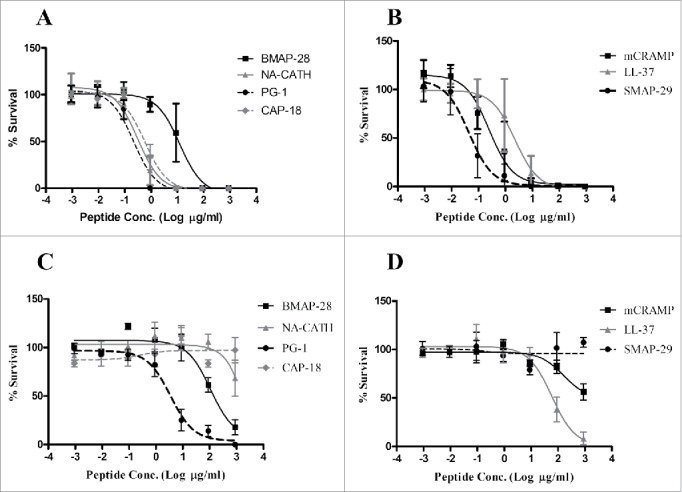
Antimicrobial peptides exert activity against vegetative bacilli and endospores. *B. anthracis* Sterne bacilli (A, B) or spores (C, D) were incubated for 3 h with a range of the log_10_ peptide concentrations in 10 mM phosphate buffer and percent (%) survival was determined (EC_50_). Standard deviations of the mean are shown on each graph as error bars.

**Table 1.** Activity of Antimicrobial peptides against vegetative *B. anthracis.* Antibacterial and Sporicidal activity of CAMPs against *B. anthracis* Sterne strain vegetative activity.

	EC_50_ (95% Confidence Interval)		Sporicidal EC_50_ (95% Confidence Interval)		MIC	
Peptide	(µg/ml)	(µM)	(µg/ml)	(µM)	(µg/ml)	(µM)
Ciprofloxacin	0.01(0.003–0.06)	0.02 (0.01–0.18)	70.8 (28.7–175)	>100	0.01	0.04
SMAP-29	0.03 (0.01–0.08)	0.01 (0.003–0.025)	>100	>100	8	2.46
NA-CATH	0.29 (0.21–0.45)	0.07 (0.05–0.11)	>100	>100	16	3.83
PG-1	0.22 (0.14–0.32)	0.10 (0.06–0.15)	3.41 (1.16–10.1)	1.58 (0.54–4.63)	16	7.41
D-LL-37	0.13 (0.07–0.34)	0.03 (0.02–0.08)	81.8 (41.8–160)	18.2 (9.30–35.6)	32	7.12
LL-37	0.22 (0.11–0.47)	0.05 (0.02–0.10)	51.1 (23.9–109)	11.4 (5.32–24.3)	32	7.12
mCRAMP	0.23 (0.14–0.43)	0.06 (0.04–0.11)	>100	>100	64	16.5
CAP-18	0.31 (0.24–0.95)	0.07 (0.05–0.21)	>100	>100	>150	>150
BMAP-28	7.89 (3.41–20.3)	2.52 (1.09–6.48)	82.1 (40.8–165)	26.2 (13.0–52.7)	>150	>150
Scrambled LL-37	>100	>100	>100	>100	>150	>150

Six of the cathelicidins demonstrated significant MIC activity (Table 1), with only CAP-18 and BMAP-28 found to be inactive. The range of MICs for active peptides was from 8–64 µg/ml (Table 1). The most effective peptide against *B. anthracis* was SMAP-29, the sheep myeloid antimicrobial peptide (MIC = 8 µg/ml). Ciprofloxacin, an antibiotic used clinically to treat anthrax infections in humans, was used as a positive control and demonstrated a MIC of 0.01 µg/ml, similar to published results.^31^ The lack of activity of CAP-18 and BMAP-28 under MIC conditions was notable, as other cathelicidins were active. The overall lack of *in vitro* activity of BMAP-28 against the vegetative cells was interesting, given that this peptide has activity against other organisms.^32-34^

Inoculation by *B. anthracis* endospores causes anthrax in mammalian hosts, thus we also examined the sporicidal activity of these peptides (Table 1). While all peptides had various killing activity against *B. anthracis* bacilli only 3 peptides had activity against *B. anthracis* spores; PG-1, BMAP-28 and LL-37 (Fig. 1C, D). PG-1 had the strongest activity in alignment with published reports of PG-1 being capable of killing spores before vegetative outgrowth.^12^ We demonstrated sporicidal activity of PG-1 with an EC_50_ of 1.58 µM (95% CI: 0.54 µM–4.63 µM). Less effective were the human cathelicidin LL-37 with an EC_50_ of 11.4 µM (95% CI: 5.32 µM–24.3 µM) and bovine cathelicidin BMAP-28 with an EC_50_ of 26.2 µM (95% CI: 13.0 µM–52.7 µM). It was determined that *B. anthracis* Sterne strain spores do not germinate after 3 hrs incubation in 10 mM phosphate buffer (data not shown).

For characterization of the *in vivo* invertebrate model *Galleria mellonella*, we first performed a standard kill curve to determine the lethal dose of *B. anthracis* Sterne strain injected into the hemocoel of the larvae (Fig. S1). *G. mellonella* larvae were injected with 10 µl of various concentrations of *B. anthracis* spores or vegetative cells and the amount that kills all larvae by 48 h was used as the LD_99_ for future experiments. It was found that 10^5^ spores were sufficient to cause death of all larvae by 48 h and this was used as our infectious dose for future experiments (LD_50_ = 3 × 10^3^) (Fig. S1A). Vegetative cells were also tested and the LD_99_ was determined to be 10^9^ cells, much higher than that for spores (LD_50_ = 10^7^) (Fig. S1B). As the usual infecting form is spores,^26^ we infected with spores for these *in vivo* experiments. Each experiment was performed with 10 randomly selected *G. mellonella* larvae and each experiment was performed 3 times with a representative experiment shown.

Waxworms were infected with 1 × 10^5^ spores per larvae *B. anthracis* Sterne spores and then treated with a single injection of 10 µg (in 10 µl) of peptide injected into the hemocoel 1 h after the infection. Controls were treated with PBS or ciprofloxacin (Fig. 2). The negative control group (PBS-treated) did not survive past 48 h. An antibiotic control was used to verify the performance of this *in vivo* model (Fig. 2A). Ciprofloxacin (1 µg/10 µl) was injected one time only into the hemocoel to treat *G. mellonella* infected with the *B. anthracis* Sterne spores, and was able to rescue 100% of infected waxworms (1 µg of ciprofloxacin per larva following 3 h of infection). As another control, we used scrambled LL-37, which is a peptide that has the same molecular weight, amino acids and charge but the order of the amino acids is changed. This control also did not survive past 48 h, consistent with our previous findings that scrambled LL-37 peptide does not confer antibacterial activity^9^ (Fig. 2A).

**Figure 2.**
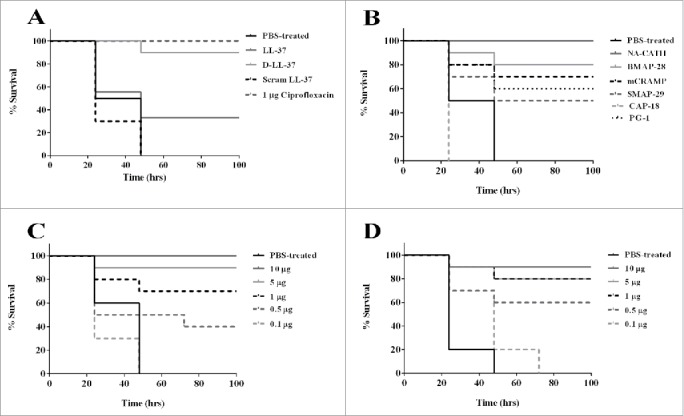
Antimicrobial peptide treatment of *B. anthracis* Sterne spore challenge of *G. mellonella. Galleria mellonella* survival curve was performed for *B. anthracis* Sterne spore infected waxworms and treated with (A) LL-37, D-LL-37 and scrambled LL-37; (B) PBS-treated, NA-CATH, BMAP-28, mCRAMP, SMAP-29, CAP-18 and PG-1; (C) dose dependence in NA-CATH and (D) dose dependence in D-LL-37. Significant rescue was observed for all peptides except CAP-18 and scrambled LL-37 while NA-CATH and D-LL-37 had survival that was statistically similar to that of the antibiotic control ciprofloxacin. For dose dependence, both peptides were able to rescue a portion *G. mellonella* at peptide concentrations as low as 0.5 µg per larva in 10 µl. NA-CATH was able to rescue a statistically similar number of larvae as ciprofloxacin at 5 µg per larvae while D-LL-37 was able to rescue a statistically similar population as ciprofloxacin with 1 µg per larvae. Kaplan-Meier statistics was performed to determine statistical significance of peptide treated versus PBS treated and p-values are shown in Table S2.

The experimental groups treated with various CAMPs demonstrated a prolonged survival time (p<0.05) for all cathelicidin peptides except CAP-18. Peptide NA-CATH treated waxworms demonstrated 100% survival and peptide D-LL-37 treated waxworms demonstrated 90% survival, which is similar to that of the antibiotic control ciprofloxacin (p > 0.05), according to Kaplan-Meier statistics estimator.^35^ Other peptides BMAP-28, mCRAMP, SMAP-29, PG-1 and LL-37 demonstrated some rescue of *B. anthracis-*infected *G. mellonella*; however, the survival was statistically different and less than that of the antibiotic control ciprofloxacin (p < 0.05) (Fig. 2A,B). Of interest, BMAP-28 treatment led to significant rescue, despite its poor *in vitro* performance against vegetative bacilli.

Peptides NA-CATH and D-LL-37, which were highly effective and comparable to ciprofloxacin in survival, were further analyzed to determine the lowest peptide concentration required for rescue of *B. anthracis* infected *G. mellonella* (Fig. 2C,D). For peptide NA-CATH, 5 µg (in 10 µl) of peptide per larva (approx. 0.3 g each) demonstrated high levels of survival (statistically similar to the antibiotic control, p < 0.001) while treatment with as low as 0.5 µg of peptide per larva still rescued a significant portion of the population. Peptide D-LL-37 rescued *B. anthracis-*infected *G. mellonella* at 1 µg of peptide per larva, (statistically similar to the antibiotic control, p < 0.001) while doses as low as 0.5 µg peptide per larva still rescued a significant portion of the population (Fig. 2C,D) (p < 0.001).

Cytotoxicity and hemolysis testing was performed for these peptides (Fig. S2). Peptide concentrations used for these experiments were 100 µg/ml, 100x higher than the concentration being used to treat *B. anthracis* infected *G. mellonella*. Defibrinated sheep blood was used for hemolytic testing and no peptide demonstrated statistically significant hemolytic activity (p < 0.001), consistent with our previous result for NA-CATH.^5^ For cytotoxicity testing, A549 human lung epithelial cells were used as they have been previously shown to be a target of internalization by *B. anthracis*.^36^ A549 cells treated with 100 µg/ml of all peptides demonstrated statistically similar cell survival (p < 0.001) compared with the PBS-treated control, while all were different from the Triton X treated control (p < 0.001), which represented 100% lysis. From these experiments, we conclude that the concentrations we were using to treat *B. anthracis* infected *G. mellonella* were not causing cytotoxicity. We also performed toxicity testing of peptides injected into the larvae. We found that peptide-only injections did not adversely affect waxworm survival (data not shown).

This study established an *in vivo* model for *B. anthracis* infection in the waxworm that may be useful for screening and testing novel antibiotics as they are developed and may enable down-selection before murine testing. This model involves injecting *B. anthracis* spores into the hemocoel of the *G. mellonella* via a pro-leg, followed 3 h later by injection of the potential therapeutic in the opposite pro-leg. This *in vivo* model allows for time-efficient and inexpensive experiments to be performed when testing a large panel of therapeutics, such as was done in this study, where the large number of in vitro active compounds would take considerable time and resources in a murine model. Other invertebrate models do exist for *B. anthracis* infection; however, they use other routes of infection such as gastrointestinal in the cases of *G. mellonella* and *C. elegans*.^37,38^ In the case of our *G. mellonella* model, we are able to test therapeutic agents against hemocoel anthrax infections, which may model disseminated anthrax. While the number of treatments can be adjusted to suit experimental needs,^8-10,16^ in this case we tested a single treatment of 10 ug of peptide per larvae injected into the hemocoel. This assay suggests that the survival-promoting peptides are strongly active to affect waxworm survival outcomes with a single treatment dose. This model enabled us to down-select from 7 CAMPS with *in vitro* activity for *B. anthracis* to 2 CAMPs with strong *in vivo* activity (D-LL-37 and NA-CATH), and 4 CAMPs (BMAP-28, mCRAMP, SMAP-29, PG-1) with moderate survival between 40–60%.

Protegrin-1 (PG-1), which was previously shown to be sporicidal, was able to rescue 60% of the waxworms in a single treatment of 10 ug (23 µM) of PG-1 peptide in our model. This compares with the published results in mice that treatment with 50 uM PG-1 given subcutaneously 4 h post infection results in 80% survival,^12^ further supporting the relevance of our *G. mellonella* model.

Our test panel of CAMPs represents a diverse set of cathelicidin peptides that could potentially exert an antimicrobial effect upon *B. anthracis*. This work examined these antibacterial abilities against *B. anthracis* Sterne, 6 of which had MIC activity, and the snake-derived cathelicidin peptide, NA-CATH, rescued 100% of waxworms following *B. anthracis* infection. Our model corroborated published data with PG-1 demonstrating activity against both vegetative bacilli and spores while LL-37 and mCRAMP were antimicrobial but not effective against the spores.^12,13^ Additionally, we have shown that these peptides were not cytotoxic, hemolytic or toxic.

*In vivo* experiments were performed in *G. mellonella* as a new *in vivo* model organism for *B. anthracis* infection. A large difference was observed between the infectious dose of vegetative bacilli and spores in the *G. mellonella* model (Fig. S1A,B). Spores have an exosporium that protects them from toxic molecules such as host-derived proteases and lysozyme, while vegetative bacterial cells would be more susceptible to assault from these molecules. Thus, in our model we used the spores to initiate the infection, although we demonstrated that waxworms are also susceptible to infection by vegetative cells, and that vegetative cells are susceptible to these CAMPs.

Both host-derived and pathogen-expressed proteases are known to play a large role in *B. anthracis* infection.^39^ We observed a significant difference (p<0.001) between the D- and L-enantiomers (90% vs. 30% survival) of LL-37 treatment (Fig. 2A) indicating that the D-enantiomer, which is resistant to protease degradation,^9^ is superior to its L-enantiomer in *in vivo* performance, although the *in vitro* MICs are statistically identical. This result agrees with our previously published results demonstrating a survival advantage for protease-resistant peptide D-LL-37 against *Pseudomonas aeruginosa* infection in *G. mellonella*.*^9^*

*G. mellonella* has numerous features that make them an appropriate surrogate for mammalian infection, including that: (1) as insects they are invertebrates and do not need IACUC approval, (2) they can be incubated at 37°C, (3) they can be easily injected *via* the prolegs, (4) they are ethically acceptable, and (5) have been developed as an infection model for several other bacteria. Additionally, the insect immune system is related to that of mammals.^40^ Insects have phagocytic cells that engulf bacteria and produce bactericidal compounds.^41^ Other advantages that we are continuing to explore in this model include performing synergy experiments with antibiotics, which can be easily achieved in this *in vivo* model. This study establishes *G. mellonella* as a useful new *in vivo* model for rapidly and efficiently testing potential therapeutic agents against *B. anthracis* infection.

## Supplementary Material

KVIR_S_1293227.zip
