# Supplementary material for: Cathelicidin peptide rescues G. mellonella infected with B. anthracis
Source: Virulence. 2017 Mar 8;9(1):287–93. doi: 10.1080/21505594.2017.1293227 (PMC6104695; doi:10.1080/21505594.2017.1293227)
Supplement: KVIR_S_1293227.zip [file kvir-09-01-1293227-s001.zip › KVIR_S_1293227.docx]

**Supplementary Materials**

**Supplemental Table Legends**

**Supplemental Table 1:** Amino acid sequences and MW of antimicrobial peptides tested.

**Supplemental Table 2:** Percent survival and p-values of peptide treatment versus PBS-treated of *B. anthracis* infected *G. mellonella* larvae.

**Supplemental Figure Legends**

**Supplemental Figure 1: Infection of *G. mellonella* with *B. anthracis* Sterne spores and vegetative bacilli.** *Galleria mellonella* survival curve was performed with waxworms injected with *B. anthracis* Sterne spores (A) and *B. anthracis* Sterne vegetative bacilli (B). *B. anthracis* Sterne (spores or bacilli) were injected into the left proleg and monitored for five days. Injection volume was always 10 µl.

**Supplemental Figure 2: Cytotoxicity and hemolytic activity of antimicrobial peptides tested in this study.** (A) Cytotoxicity was not observed for any peptide in the panel when tested against A549 cells at 100 µg/ml. (B) No hemolytic activity was observed for any peptide at 100 µg/ml in the panel when tested against defibrinated sheep blood. Student’s t-test was performed and showed there was no statistical hemolytic activity due to these antimicrobial peptides. Standard deviations of the mean are shown on each graph as error bars.

**Supplemental Table 1. Amino acid sequences and MW of antimicrobial peptides and antibiotics tested**

| **Peptide** | **MW** | **Sequence** | **Reference** |
| --- | --- | --- | --- |
| SMAP-29 | 3256 | RGLRRLGRKIAHGVKKYGPTVLRIIRIAG | ^1^ |
| NA-CATH | 4175.22 | KRFKKFFKKLKNSVKKRAKKFFKKPKVIGVTFPF | ^2^ |
| PG-1 | 2160.63 | RGGRLCYCRRRFCVCVGR | ^3^ |
| D-LL-37 | 4493.3 | LLGDFFRKSKEKIGKEFKRIVQRIKDFLRNLVPRTES | ^4^ |
| LL-37 | 4493.3 | LLGDFFRKSKEKIGKEFKRIVQRIKDFLRNLVPRTES | ^5^ |
| mCRAMP | 3878.66 | GLLRKGGEKIGEKLKKIGQKIKNFFQKLVPQPEQ | ^6^ |
| CAP-18 | 4433.45 | GLRKRLRKFRNKIKEKLKKIGQKIQGLLPKLAPRTDY | ^7^ |
| BMAP-28 | 3131.89 | GGLRSLGRKILRAWKKYGPIIVPIIRIG | ^8^ |
| Scrambled LL-37 | 4493.3 | GLKLRFEFSKIKGEFLKTPEVRFRDIKLKDNRISVQR | ^4^ |
| Ciprofloxacin | 331.3 | N/A |  |

**Supplemental Table 2.** Percent survival and p-values of peptide treatment versus PBS-treated of *B. anthracis* infected *G. mellonella* larvae

| **Peptide** | **Survival (%)** | **p-value** |
| --- | --- | --- |
| SMAP-29 | 50 | 0.0307 |
| NA-CATH | 100 | <0.0001 |
| PG-1 | 60 | 0.0075 |
| D-LL-37 | 90 | <0.0001 |
| LL-37 | 40 | 0.1009 |
| mCRAMP | 70 | 0.0032 |
| CAP-18 | 0 | 1.000 |
| BMAP-28 | 80 | 0.0005 |
| Scrambled LL-37 | 0 | 0.3736 |
| Ciprofloxacin | 100 | <0.0001 |

**Supplemental Methods**

**Bacterial preparation**

*B. anthracis* Sterne strain was obtained from the Colorado Serum Co. (Boulder, CO). *B. anthracis* spores were prepared as described previously ^9^. Bacteria were grown in LB overnight in a shaking incubator (37^°^C) and due to the ability of *Bacillus* to grow in chains, bacterial growth was determined by measuring OD_600_, and glycerol stocks prepared and frozen at -80^o^C.

**Antimicrobial Peptides and Ciprofloxacin**

Peptides were synthesized by ChinaPeptides, Inc (Shanghai, China) using Fmoc chemistry. Peptides were provided at >95% purity as TFA salts. Peptide concentration and purity were confirmed with RP-HPLC and ESI-MS. A scrambled version of LL-37 was used as a control for the presence of peptide with the same net charge and amino acid composition, but different sequence (**Table S1**). Peptides were reconstituted in PBS for injections into *G. mellonella* larvae.

Ciprofloxacin was prepared by dissolution into an acidic aqueous solution at a concentration of 1 mg/mL and then titrated up to a pH of 7.4, similar to that of PBS.

**Antibacterial activity assay**

The antimicrobial (EC_50_) activity of the peptides against *B. anthracis* was determined for both vegetative bacilli ^10^ as well as spores as previously described ^11^. Briefly, 1x10^5^ CFU per well (100µl volume),from the frozen glycerol stock prepared as described above, were incubated for 3 h with different peptide (peptide was reconstituted in PBS) concentration in 10 mM sodium phosphate buffer, pH 7.4, in 96-well plates (Corning). Serial dilutions of each peptide were performed in the 96-well plate and then incubated with the bacteria. Each experiment repeated three independent times. Bacterial survival was then determined by serial dilutions and plating in triplicate on LB plates which were incubated 24 h at 37^°^C and CFUs counted. Bacterial survival at each peptide concentration was calculated based on the ratio of the number of colonies on each experimental plate and the average number of colonies on the control plate of cultures lacking peptide. The peptide concentration required to inhibit 50% of growing colonies was determined by plotting the percent killing as a function of the log of peptide concentration (ug/ml) and fitting the data using GraphPad Prism 6.0. EC_50_ in ug/ml or uM is reported including the 95% confidence interval, which represents p<0.05.

It was determined that *B. anthracis* Sterne strain spores do not germinate after 3 hrs incubation in 10 mM phosphate buffer (data not shown), which was determined by light microscopy.

**Minimum inhibitory concentration (MIC) assay**

Broth microdilution assays were performed in 96-well tissue culture plates (Corning) and following Clinical and Laboratory Standards Institute (CLSI) guidelines^12^. Briefly, 1x10^5^ CFUs of vegetative bacteria per well were incubated with varying concentrations of peptide in 200 µl of Mueller Hinton II Broth (Cation-Adjusted, BBL L007475). 96-well plates were incubated at 37^°^C under static conditions for 24 h and then OD_600_ measured. The MIC refers to the lowest concentration of peptide that cleared >90% of the optical density.

***G. mellonella* infection and treatment**

*G. mellonella* were obtained at the larval stages from Vanderhorst Whole Sale (Saint Mary’s, OH) and stored at room temperature until injection. 10 caterpillars with a weight range of 0.30-0.35 g were randomly assigned to each group and each of the three replicates per experiment were from different batches of *G. mellonella*. Unless otherwise stated, *G. mellonella* larvae were infected with 1x10^5^ *B. anthracis* Sterne spores by injection into the left proleg, following our previously described protocols for other bacteria ^13-15^. After 1-h incubation at 37^°^C, larvae were treated with 10 µg of antimicrobial peptide, PBS or Ciprofloxacin into the right proleg as previously described ^16^. Survival was recorded every 24h for 5 days. Injection volumes were always 10 µl: this volume was measured out by pipette and then aspirated and injected into *G. mellonella* larvae from a 27G needle and syringe (BD Biosciences). Each of the three replicates for *G. mellonella* studies were statistically similar and a representative experiment is shown.

For toxicity testing of peptides, 10 µg of peptide was injected into uninfected *G. mellonella* larvae as described above and monitored for 10 days. No statistically significant toxicity effect was observed (data not shown).

**Cytotoxicity assays**

Cytotoxicity assays were performed using the Vybrant MTT Cell Proliferation Assay Kit (Life Technologies) according to manufacturer’s instructions ^17^. Assays were performed using human epithelial lung carcinoma line A549 (ATCC CCL-185), which was maintained in Dulbecco’s Minimal Essential Medium (Life Technologies) prepared with 10% FBS. 100 µg/ml of peptide was used for each experimental well in 96-well tissue culture plates (Corning). Each experiment was performed three times in triplicate for a total n=9.

**Hemolysis assays**

Hemolysis assays were performed as previously described ^2^, using defibrinated sheep’s blood (Remel Microbiology). Peptide was reconstituted from lyophilized stocks with PBS at a concentration of 200 µg/ml. Defibrinated sheep’s blood was purified by centrifugation at 1600 rpm for 10 min and supernatant removed contaminants in the original suspension. A 2% blood suspension was incubated with a final concentration of 100 µg/ml of peptide at 37^°^C for 1 h. After incubation, whole suspension results were read on a spectrophotometer at 540 nm. Each experiment was performed three times in triplicate for a total n=9. A 100% hemolysis control was included in which defibrinated sheep blood was suspended in water, causing 100% lysis. PBS alone was used as a negative control (0% hemolysis).

**Statistical analysis**

Antimicrobial and sporicidal EC_50_ assays were performed in triplicate with n=3 for each experiment. Standard deviations of the mean are shown on each graph as error bars. Also, 95% confidence intervals are provided for EC_50_ assays to demonstrate statistical overlap of data. For the MIC assay, cytotoxicity assay and hemolytic assays Student’s t-test was performed and p-values of p<0.05 were considered statistically different.

For *G. mellonella in vivo* infection and treatment experiments Kaplan-Meier statistics was performed to determine statistical significance (GraphPad Prism 6.0). The p-values of peptide treated versus PBS treated are shown in **Table S2**. The survival curves were performed with an n=10 and repeated for a total of three independent experiments.

**Supplemental References**

1. Skerlavaj B, Benincasa M, Risso A, Zanetti M, Gennaro R. SMAP-29: a potent antibacterial and antifungal peptide from sheep leukocytes. FEBS Lett 1999; 463:58-62.

2. de Latour FA, Amer LS, Papanstasiou EA, Bishop BM, van Hoek ML. Antimicrobial activity of the Naja atra cathelicidin and related small peptides. Biochemical and biophysical research communications 2010; 396:825-30.

3. Donati M, Di Francesco A, Gennaro R, Benincasa M, Di Paolo M, Shurdhi A, et al. Increasing effect of a high dose of PG-1 peptide on the infectivity of Chlamydophila abortus. FEMS Immunol Med Microbiol 2010; 59:221-2.

4. Dean SN, Bishop BM, van Hoek ML. Natural and synthetic cathelicidin peptides with anti-microbial and anti-biofilm activity against Staphylococcus aureus. BMC microbiology 2011; 11:114.

5. Turner J, Cho Y, Dinh NN, Waring AJ, Lehrer RI. Activities of LL-37, a cathelin-associated antimicrobial peptide of human neutrophils. Antimicrobial agents and chemotherapy 1998; 42:2206-14.

6. Gallo RL, Kim KJ, Bernfield M, Kozak CA, Zanetti M, Merluzzi L, et al. Identification of CRAMP, a cathelin-related antimicrobial peptide expressed in the embryonic and adult mouse. The Journal of biological chemistry 1997; 272:13088-93.

7. Hirata M. [Anti microbial, LPS-neutralizing protein (CAP 18) and host defense]. Tanpakushitsu Kakusan Koso 2001; 46:575-81.

8. Risso A, Braidot E, Sordano MC, Vianello A, Macri F, Skerlavaj B, et al. BMAP-28, an antibiotic peptide of innate immunity, induces cell death through opening of the mitochondrial permeability transition pore. Mol Cell Biol 2002; 22:1926-35.

9. Popov SG, Villasmil R, Bernardi J, Grene E, Cardwell J, Wu A, et al. Lethal toxin of Bacillus anthracis causes apoptosis of macrophages. Biochemical and biophysical research communications 2002; 293:349-55.

10. Luna VA, King DS, Gulledge J, Cannons AC, Amuso PT, Cattani J. Susceptibility of Bacillus anthracis, Bacillus cereus, Bacillus mycoides, Bacillus pseudomycoides and Bacillus thuringiensis to 24 antimicrobials using Sensititre automated microbroth dilution and Etest agar gradient diffusion methods. The Journal of antimicrobial chemotherapy 2007; 60:555-67.

11. Lisanby MW, Swiecki MK, Dizon BL, Pflughoeft KJ, Koehler TM, Kearney JF. Cathelicidin administration protects mice from Bacillus anthracis spore challenge. Journal of immunology 2008; 181:4989-5000.

12. (CLSI) CLSI. Methods for Dilution Antimicrobial Susceptability Tests for Bacteria that grow aerobically; Approved Standard - Tenth Edition. CLSI Document M07-A10. Wayne, PA: Clinical Laboratory Standards Institute, 2015.

13. McKenney ES, Sargent M, Khan H, Uh E, Jackson ER, San Jose G, et al. Lipophilic prodrugs of FR900098 are antimicrobial against Francisella novicida in vivo and in vitro and show GlpT independent efficacy. PloS one 2012; 7:e38167.

14. Dean SN, Bishop BM, van Hoek ML. Susceptibility of Pseudomonas aeruginosa Biofilm to Alpha-Helical Peptides: D-enantiomer of LL-37. Frontiers in microbiology 2011; 2:128.

15. Aperis G, Fuchs BB, Anderson CA, Warner JE, Calderwood SB, Mylonakis E. Galleria mellonella as a model host to study infection by the Francisella tularensis live vaccine strain. Microbes Infect 2007; 9:729-34.

16. Georgi E, Schacht E, Scholz HC, Splettstoesser WD. Standardized broth microdilution antimicrobial susceptibility testing of Francisella tularensis subsp. holarctica strains from Europe and rare Francisella species. J Antimicrob Chemother 2012; 67:2429-33.

17. Barksdale SM, Hrifko EJ, Chung EM, van Hoek ML. Peptides from American alligator plasma are antimicrobial against multi-drug resistant bacterial pathogens including Acinetobacter baumannii. BMC microbiology 2016; 16:189.
